# ﻿Three new species of *Carlosrosaea* (Trimorphomycetaceae, Tremellales) from China

**DOI:** 10.3897/mycokeys.119.151751

**Published:** 2025-07-01

**Authors:** Wan-Li Gao, Chun-Yue Chai, Qiu-Hong Niu, Feng-Li Hui

**Affiliations:** 1 School of Life Science, Nanyang Normal University, Nanyang 473061, China Nanyang Normal University Nanyang China; 2 Research Center of Henan Provincial Agricultural Biomass Resource Engineering and Technology, Nanyang Normal University, Nanyang 473061, China Nanyang Normal University Nanyang China

**Keywords:** Basidiomycetes, biodiversity, foliicolous yeast, phylogenetic analysis, taxonom

## Abstract

*Carlosrosaea* is a genus of plant-associated yeasts within the family Trimorphomycetaceae of the order Tremellales. Currently, eight species have been described and accepted as members of the genus *Carlosrosaea*. In this study, six *Carlosrosaea* strains were isolated from the surface of leaves of the genera *Camellia*, *Distylium*, *Glechoma*, and *Glochidion*, collected in Fujian, Guizhou, Hainan, and Henan provinces. Based on phylogenetic analyses and phenotypic characterization, these strains were identified as three new *Carlosrosaea* species: *C.camelliae***sp. nov.** (holotype CICC 33566^T^), *C.glechomae***sp. nov.** (holotype CICC 33632^T^), and *C.wuzhiensis***sp. nov.** (holotype GDMCC 2.526^T^). Descriptions, illustrations, and phylogenetic data of these new taxa are provided. This study enhances knowledge of *Carlosrosaea* species diversity in China and provides a foundation for future taxonomic and ecological research.

## ﻿Introduction

The genus *Carlosrosaea* was established by Liu et al. (2015) to include *Bulleravrieseae* Landell, L.R. Brandão, Safar, F.C.O. Gomes, C.R. Félix, A.R.O. Santos, D.M. Pagani, J.P. Ramos, Broetto, T. Mott, Vainstein, P. Valente & C.A. Rosa. Subsequently, two additional species, *C.hohenbergiae* C.R. Félix, H.M.C. Navarro, Paulino, Broetto & Landell and *C.aechmeae* C.R. Félix, H.M.C. Navarro, Paulino, Broetto & Landell, were described from bromeliads in Brazil (Félix et al. 2017). More recently, five more species—*C.foliicola* Q.M. Wang, F.Y. Bai & A.H. Li, *C.simaoensis* Q.M. Wang, F.Y. Bai & A.H. Li, *C.betulae* Q.M. Wang, *C.rhododendri* Q.M. Wang, and *C.yunnanensis* Q.M. Wang—were identified from plant leaves in China (Li et al. 2020; Jiang et al. 2024). Additionally, sequence data from public databases indicate the existence of more than nine putative novel species within the genus (Li et al. 2020; Jiang et al. 2024).

All known species of *Carlosrosaea* exhibit an asexual morph, characterized by polar budding as the primary mode of reproduction (Landell et al. 2015; Félix et al. 2017; Li et al. 2020; Jiang et al. 2024). Most species do not produce hyphae or ballistoconidia, although some are capable of forming pseudohyphae (Li et al. 2020). Physiologically, members of the genus lack fermentative ability but can assimilate a wide range of carbon sources, excluding methanol and hexadecane. Nitrate utilization has been observed in some species (Liu et al. 2015), while the production of starch-like compounds is generally negative (Li et al. 2020; Jiang et al. 2024). Notably, recent studies have indicated that *C.vrieseae* possesses plant growth-promoting traits, including phosphate solubilization, siderophore secretion, and indole-3-acetic acid (IAA) synthesis, suggesting potential applications as a biofertilizer (Marques et al. 2021).

Species of the genus *Carlosrosaea* are considered epiphytic yeasts associated with plants, mostly with flowers (Félix et al. 2017), leaves (Landell et al. 2015; Félix et al. 2017; Li et al. 2020; Jiang et al. 2024), and phytotelmata (Landell et al. 2015; Marques et al. 2021). *Carlosrosaea* species have also been reported from soil (Félix et al. 2024).

To date, eight species of *Carlosrosaea* have been reported worldwide, all originally described from Asia and South America (Félix et al. 2024). In China, five species have been described so far (Li et al. 2020; Jiang et al. 2024). Most of these species are found in temperate and subtropical regions of China, while a few occur in tropical areas. In our survey over the past three years, phylogenetic analyses of combined ITS and LSU sequence data, along with phenotypic characterization of six *Carlosrosaea* isolates obtained from plant leaves across different locations in China, led to the identification of three new species: *Carlosrosaeacamelliae* sp. nov., *Carlosrosaeaglechomae* sp. nov., and *Carlosrosaeawuzhiensis* sp. nov., which are described here.

## ﻿Materials and methods

### ﻿Sample collection and yeast isolation

A total of 49 leaf samples were collected from four natural forests in China: nine from a subtropical semi-deciduous forest in Fujian (25°7'N, 118°44'E), seven from a subtropical semi-deciduous and deciduous mixed forest in Guizhou (25°7'N, 107°2'E), 15 from a subtropical deciduous forest in Henan (32°45'N, 113°30'E), and 18 from tropical rainforests in Hainan (18°19'N, 109°9'E). Samples were placed in sterile, self-sealing plastic bags for transport and stored at 5 °C until processing. Yeast strains were isolated from leaf surfaces using a spore drop method described by Toome et al. (2013). Fresh leaves were cut into small pieces and affixed to the inner lid of a Petri dish using a thin layer of petroleum jelly. The Petri dish contained yeast extract–malt extract (YM) agar medium (0.3% yeast extract, 0.3% malt extract, 0.5% peptone, 1% glucose, and 2% agar), supplemented with 0.01% chloramphenicol to inhibit bacterial growth. Plates were incubated at 20 °C and monitored daily for colony development. Emerging yeast colonies were streaked onto fresh YM agar plates for purification. Purified strains were suspended in 20% (v/v) glycerol and stored at −80 °C for long-term preservation. Cultures of all obtained isolates were preserved at the Microbiology Lab, Nanyang Normal University (NNUML), Henan, China.

### ﻿Phenotypic characterization

Morphological, physiological, and biochemical characteristics were assessed following standardized methods established by Kurtzman et al. (2011). Colony morphology was observed on YM agar after 7 days of incubation at 20 °C. Cell morphology was examined in YM broth after 3 days of incubation at 20 °C using a LEICA DM2500 microscope with LAS V4.13 software. Ballistoconidium-forming activity was assessed using the inverted-plate method on cornmeal agar (CMA: 2.5% cornmeal infusion and 2% agar) at 17 °C, as described by do Carmo-Sousa and Phaff (1962). After 3 to 14 days, discharged spores were collected on a glass slide and examined microscopically. The potential presence of a sexual cycle was investigated by culturing strains on CMA, potato dextrose agar (PDA: 20% potato infusion, 2% glucose, and 2% agar), and V8 agar (10% V8 juice and 2% agar). Strains were inoculated individually and in mixed cultures, with incubation at 20 °C for up to two months. Observations were conducted at two-week intervals (Li et al. 2020). Glucose fermentation was tested in liquid medium using Durham fermentation tubes. Carbon and nitrogen assimilation were assessed in liquid media, with starved inoculum employed for nitrogen assimilation tests (Kurtzman et al. 2011). Growth at different temperatures (15, 20, 25, 30, 35, and 37 °C) was evaluated on YM agar plates. Proposed names and descriptions were deposited in the MycoBank database (http://www.mycobank.org; 25 February 2025).

### ﻿DNA extraction, PCR amplification, and sequencing

Genomic DNA was extracted from actively growing yeast cells cultured on YM agar using the Ezup Column Yeast Genomic DNA Purification Kit, according to the manufacturer’s protocol (Sangon Biotech Co., Shanghai, China). The ITS region and the D1/D2 domain of the LSU rRNA gene were amplified and sequenced using primers ITS1/ITS4 (White et al. 1990) and NL1/NL4 (Kurtzman and Robnett 1998), respectively.

PCR amplification was performed in a 25 µL reaction volume consisting of 9.5 µL ddH_2_O, 12.5 µL of Taq 2 × PCR Master Mix with blue dye (Sangon Biotech Co., Shanghai, China), 1 µL of DNA template, and 1 µL of each primer. Amplification was conducted using an AB 2720 thermal cycler (Applied Biosystems, Foster City, California, USA), with the following program: 98 °C for 2 min; 35 cycles of 98 °C for 10 s, 52 °C for 10 s, and 72 °C for 15 s; followed by a final extension at 72 °C for 5 min (Chai et al. 2024). PCR products were verified by electrophoresis on a 1% (w/v) agarose gel. Positive reactions showing a bright single band were purified and sequenced by Sangon Biotech (Shanghai) Co., Ltd. (Shanghai, China). The identity and accuracy of each sequence were confirmed by comparison with sequences in the GenBank database. Sequence assembly was performed using BioEdit v.7.1.3.0 (Hall 1999). All newly generated sequences were deposited in the GenBank database (https://www.ncbi.nlm.nih.gov/genbank/).

### ﻿Phylogenetic analyses

For phylogenetic analyses, 12 newly obtained ITS and LSU sequences from this study, along with 50 sequences retrieved from the GenBank database, were included (Table 1).

**Table 1.** List of species, strains, and GenBank accession numbers of sequences used in phylogenetic analyses.

Taxon name	Strain number	Country	GenBank accession no.	
			ITS	LSU D1/D2
*Carlosrosaeaaechmeae*	CBS 14578^T^	Brazil	NR_160562	NG_064406
*Carlosrosaeabetulae*	YN35-7^T^	China	OP470240	OP470144
*Carlosrosaeabetulae*	NYNU 208206	China	MW365543	MW365542
***Carlosrosaeacamelliae***	**NYNU 223212**	**China**	**OP287964**	**OP287962**
***Carlosrosaeacamelliae***	**NYNU 223230^T^**	**China**	**OP278681**	**OP278682**
*Carlosrosaeafoliicola*	CGMCC 2.3447^T^	China	NR_174728	MK050282
***Carlosrosaeaglechomae***	**NYNU 2311170^T^**	**China**	**PP049020**	**PP033670**
***Carlosrosaeaglechomae***	**NYNU 232184**	**China**	**PV138022**	**PV138021**
*Carlosrosaeahohenbergiae*	CBS 14563^T^	Brazil	NR_159754	NG_064407
*Carlosrosaearhododendri*	JZXS7-21^T^	China	OP470238	OP470142
*Carlosrosaeasimaoensis*	CGMCC 2.3580^T^	China	NR_174729	MK050283
*Carlosrosaeavrieseae*	UFMG-CM-Y379^T^	Brazil	JX280388	JX268526
*Carlosrosaeavrieseae*	UFMG-BRO443	Brazil	JX268526	JX280388
***Carlosrosaeawuzhiensis***	**NYNU 24841^T^**	**China**	**PQ568987**	**PQ568982**
***Carlosrosaeawuzhiensis***	**NYNU 248116**	**China**	**PV637108**	**PV637107**
*Carlosrosaeayunnanensis*	YN28M1^T^	China	OP470239	OP470143
*Carlosrosaea* sp.	BSB 46	China	KX009102	KX009084
*Carlosrosaea* sp.	BSB 27	China	KX009101	KX009083
*Carlosrosaea* sp.	BM 78	Brazil	KX009086	KX009068
*Carlosrosaea* sp.	BPT 61	Brazil	KX009095	KX009077
*Carlosrosaea* sp.	BM 89	Brazil	KX009094	KX009076
*Carlosrosaea* sp.	BM 108	Brazil	KX009093	KX009075
*Carlosrosaea* sp.	BSB 17	Brazil	KX009096	KX009078
*Carlosrosaea* sp.	BSB 24	Brazil	KX009097	KX009079
*Carlosrosaea* sp.	BSS 150	Brazil	KX009099	KX009081
*Carlosrosaea* sp.	BSS 145	Brazil	KX009098	KX009080
*Carlosrosaea* sp.	BSS 158	Brazil	KX009100	KX009082
*Carlosrosaea* sp.	BM 77	Brazil	KX009085	KX009067
*Sugitazymamiyagiana*	CBS 7526^T^	Japan	NR_073237	NG_058409
*Tremellaglobispora*	CBS 6972^T^	Canada	NR_155889	NG_057771
*Tremellatropica*	CBS 8483^T^	Taiwan	NR_155938	NG_058416

Note. **^T,^** type strain. The newly generated sequences are indicated in bold.

The ITS and LSU sequences were aligned using MAFFT v.7.110 (Katoh and Standley 2013) with the G-INS-i option. Poorly aligned regions were excluded and manually adjusted using MEGA v.11 (Tamura et al. 2021). The most appropriate model of DNA substitution was determined using MEGA v. 11 (Tamura et al. 2021), and the GTR + I + G model was selected for both maximum likelihood (ML) and Bayesian inference (BI) analyses. ML analysis was performed using RAxML v.8.2.3 (Stamatakis 2014) with 1,000 rapid bootstrap (BS) replicates. BI analysis was conducted using MrBayes v.3.2.7a (Ronquist et al. 2012) via the CIPRES Science Gateway v.3.3. Six simultaneous Markov chains were run for 50 million generations, with trees sampled every 1,000 generations. The first 25% of trees were discarded as burn-in, and the remaining trees were used to estimate Bayesian posterior probabilities (BPP) for the clades.

Phylogenetic trees were visualized using FigTree v.1.4.3 (Andrew 2016), with further editing and composition conducted in Adobe Illustrator CS v.5. Branches that received BS ≥ 50% and BPP ≥ 0.95 were considered significantly supported.

## ﻿Results

### ﻿Phylogeny

**Figure 1.**
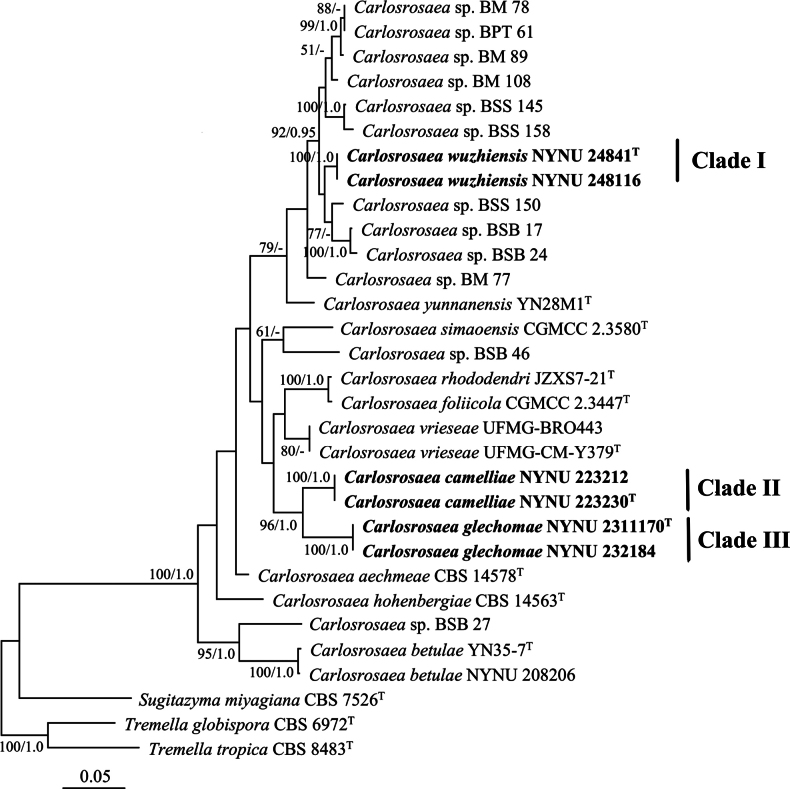
Maximum likelihood phylogenetic tree of *Carlosrosaea* generated from the combined ITS and LSU sequence data. The tree is rooted with *Tremellaglobispora* CBS 6972 and *Tremellatropica* CBS 8483. Bootstrap values (BS) ≥ 50% and Bayesian posterior probabilities (BPP) ≥ 0.95 are shown above branches. Type strain sequences are marked with (T). New species are highlighted in bold.

Phylogenetic analyses based on a combined ITS and LSU dataset was used to determine the taxonomic position of the newly isolated strains within *Carlosrosaea*. The aligned dataset was 1,053 bp in length after exclusion of poorly aligned sites, with 512 bp for ITS and 541 bp for LSU. The resulting phylogenetic tree revealed that six isolates grouped into three genetically distinct clades, each representing a putative novel species within *Carlosrosaea* (Fig. 1).

Isolates NYNU 24841 and NYNU 248116 possessed identical ITS and D1/D2 sequences, indicating conspecificity. These two isolates formed a distinct clade (Clade I), which grouped with nine unpublished sequences (BM 78, BPT 61, BM 89, BM 108, BSB 17, BSB 24, BSS 150, BSS 145, and BSS 158) from Brazil identified as *Carlosrosaea* sp., and with *C.yunnanensis* from China (Fig. 1). The two isolates differed from the nine unpublished sequences by 4–9 nucleotide (nt) substitutions (~0.7–1.6%) in the D1/D2 domain and 17–26 nt mismatches (~3.5–5.4%) in the ITS region. Additionally, they differed from their closest known relative, *C.yunnanensis*, by 9 nt substitutions (~1.6%) in the D1/D2 domain and 35 nt mismatches (~6.9%) in the ITS region. According to Vu et al. (2016), species-level thresholds for yeast identification based on barcode data from approximately 9,000 strains are 0.49% for the D1/D2 domain and 1.59% for the ITS region, consistent with previous studies (Kurtzman and Robnett 1998; Fell et al. 2000; Scorzetti et al. 2002). Therefore, the observed genetic differences support the designation of NYNU 24841 and NYNU 248116 as a novel species within *Carlosrosaea*.

Clade II, comprising strains NYNU 223230 and NYNU 223212, formed a separate branch along with Clade III, which included strains NYNU 2311170 and NYNU 232184, in the phylogenetic tree based on the combined ITS and LSU dataset (Fig. 1). The strains in Clade II had identical ITS and D1/D2 sequences, indicating conspecificity. Similarly, strains in Clade III also exhibited identical ITS and D1/D2 sequences but differed from Clade II by 10 nt substitutions (~1.8%) in the D1/D2 domain and 31 nt mismatches (~7.3%) in the ITS region. Furthermore, the four strains in Clades II and III differed from all previously described *Carlosrosaea* species by more than 13 nt substitutions (~2.3%) in the D1/D2 domain and 41 nt mismatches (~7.9%) in the ITS region. Based on the species-level thresholds proposed by Vu et al. (2016), these genetic divergences support the recognition of the four strains as two additional novel species within *Carlosrosaea*.

### ﻿Taxonomy

#### ﻿ Carlosrosaea camelliae

Taxon classificationFungiTremellalesTrimorphomycetaceae

W.L. Gao & F.L. Hui sp. nov.

C658D22A-D4F9-5022-B700-E15739CE2A00

857758

Fig. 2A, B

##### Etymology.

The specific epithet *camelliae* refers to *Camellia*, the name of the genus of the plant from which the type species was collected.

##### Type.

China• Fujian Prov.: Quanzhou City, Qingyuan Mountain, 25°7'N, 118°44'E, in the phylloplane of *Camellia* sp., March 2022, W.T. Hu and S.B. Chu, NYNU 223230 (holotype CICC 33566^T^, preserved in a metabolically inactive state; culture ex-type PYCC 9958, preserved in a viable metabolically inactive state; GenBank: OP278681, OP278682).

##### Description.

**Figure 2.**
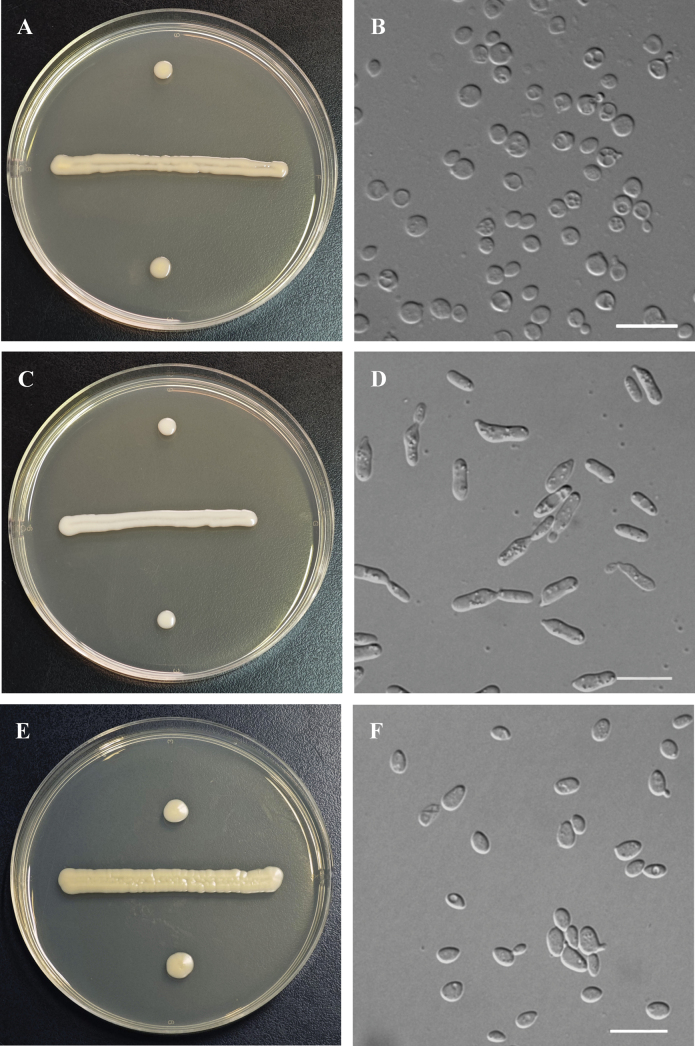
*Carlosrosaeacamelliae* sp. nov. (NYNU 223230^T^). **A** Culture on YM agar at 20 °C after 7 d; **B.** Budding cells grown in YM broth at 20 °C after 3 d. *Carlosrosaeaglechomae* sp. nov. (NYNU 2311170^T^); **C.** Culture on YM agar at 20 °C after 7 d; **D.** Budding cells in YM broth at 20 °C after 3 d. *Carlosrosaeawuzhiensis* sp. nov. (NYNU 24841^T^); **E.** Culture on YM agar at 20 °C after 7 d; **F.** Budding cells grown in YM broth at 20 °C after 3 d. Scale bars: 10 μm.

On YM agar after 7 days at 20 °C, the streak culture is white to pale-yellow, butyrous, smooth, and glossy, with an entire margin (Fig. 2A). After 3 days in YM broth at 20 °C, cells are ovoid, 2.1–4.0 × 2.6–4.9 μm, and single; budding is polar (Fig. 2B). After 1 month at 20 °C, a ring and sediment are present. In Dalmau plate culture on CMA, pseudohyphae and hyphae are not formed. Sexual structures are not observed on PDA, CMA, or V8 agar. Ballistoconidia are not produced. Glucose fermentation is absent. The following compounds are assimilated as sole carbon sources: glucose, sucrose, raffinose, melibiose, galactose, trehalose (weak), maltose (weak), melezitose, methyl-α-D-glucoside, cellobiose (weak), salicin (delayed and weak), L-sorbose, L-rhamnose (weak), D-xylose, L-arabinose (weak), D-arabinose, 5-keto-D-gluconate, D-ribose, glycerol (weak and delayed), erythritol, ribitol (delayed), galactitol, D-mannitol, D-glucitol, DL-lactate (delayed), succinate, citrate, D-gluconate, D-glucosamine (weak), N-acetyl-D-glucosamine, 2-keto-D-gluconate (weak), D-glucuronate (weak), and glucono-1,5-lactone. Inulin, lactose, methanol, ethanol, and myo-inositol are not assimilated. Nitrate, nitrite, ethylamine, L-lysine, and cadaverine are not assimilated as sole nitrogen sources. Maximum growth temperature is 30 °C. Growth on 50% (w/w) glucose-yeast extract agar is negative. Growth in vitamin-free medium is positive. Starch-like substances are not produced. Urease activity is positive. Diazonium Blue B reaction is positive.

##### Additional strain examined.

China• Fujian Prov.: Quanzhou City, Qingyuan Mountain, 25°7'N, 118°44'E, in the phylloplane of *Camellia* sp., March 2022, W.T. Hu and S.B. Chu, NYNU 223212.

##### Note.

*C.camelliae* sp. nov. is phylogenetically closely related to *C.glechomae* sp. nov., which is also described in this study, but they exhibit clear morphological and physiological differences (Table 2). Colonies of *C.camelliae* sp. nov. are white to pale yellow on YM agar, whereas those of *C.glechomae* sp. nov. are white to cream-colored. *C.camelliae* sp. nov. produces ovoid cells, while *C.glechomae* sp. nov. forms cylindrical cells. In addition, the cells of *C.camelliae* sp. nov. are shorter (2.6–4.9 μm) compared to those of *C.glechomae* sp. nov. (3.3–15 μm). Physiologically, *C.camelliae* sp. nov. differs from *C.glechomae* sp. nov. by its inability to assimilate inulin, lactose, myo-inositol, nitrate, and L-lysine, and its ability to assimilate glycerol and erythritol.

**Table 2.** Physiological and biochemical characteristics that differ between the new species and closely related species.

Characteristics	*C.wuzhiensis*	C.yunnanensis*	*C.camelliae*	*C.glechomae*
Carbon assimilation				
Inulin	–	+	–	w
Lactose	d/w	l/w	–	w
Cellobiose	+	–	w	+
L-Sorbose	d/w	–	+	d
D-Arabinose	+	–	+	+
Glycerol	d/w	–	d/w	–
Erythritol	–	w	+	–
Galactitol	d/w	–	+	w
Myo-inositol	d/w	+	–	d/w
Citrate	+	–	+	+
Nitrogen assimilation				
Nitrate	d/w	+	–	d/w
L-Lysine	+	+	–	+
Cadaverine	–	+	–	–
Growth tests				
Growth in vitamin-free medium	+	–	+	+
Growth at 25 °C	+	–	+	+

Note. + positive reaction; – negative reaction; d, delayed positive; l, latently positive; w, weakly positive. All data from this study, except* which were obtained from the original description (Jiang et al. 2024).

#### ﻿ Carlosrosaea glechomae

Taxon classificationFungiTremellalesTrimorphomycetaceae

W.L. Gao & F.L. Hui sp. nov.

A480DBCE-2149-5E8F-A773-C6DA74570F30

857759

Fig. 2C, D

##### Etymology.

The specific epithet “*glechomae*” refers to *Glechoma*, the name of the genus of the plant from which the type species was collected.

##### Type.

China• Henan Prov.: Xixia Co., Funiu Mountain, 32°45'N, 113°30'E, in the phylloplane of *Glechomalongituba* (Nakai) Kuprian, Oct 2023, S. Liu & Y.Z. Qiao, NYNU 2311170 (holotype CICC 33632^T^, preserved in a metabolically inactive state; culture ex-type PYCC 9998, preserved in a viable metabolically inactive state; GenBank: PP049020, PP033670).

##### Description.

On YM agar after 7 days at 20 °C, the streak culture is white-cream, butyrous, smooth, and glossy, with an entire margin (Fig. 2C). After 3 days in YM broth at 20 °C, cells are cylindrical, 2.6–5.7 × 3.3–15 μm, and single; budding is polar (Fig. 2D). After 1 month at 20 °C, a ring and sediment are present. In Dalmau plate culture on CMA, pseudohyphae and hyphae are not formed. Sexual structures are not observed on PDA, CMA, or V8 agar. Ballistoconidia are not produced. Glucose fermentation is absent. The following compounds are assimilated as sole carbon sources: glucose, inulin (weak), sucrose, raffinose, melibiose, galactose, lactose (weak), trehalose, maltose (weak), melezitose, methyl-α-D-glucoside (weak), cellobiose, salicin (weak), L-sorbose (delayed), L-rhamnose (weak), D-xylose, L-arabinose (weak), D-arabinose, 5-keto-D-gluconate, D-ribose, ribitol, galactitol (weak), D-mannitol (weak), D-glucitol, myo-inositol (delayed and weak), succinate, citrate, D-gluconate (weak), D-glucosamine (weak), N-acetyl-D-glucosamine (weak), 2-keto-D-gluconate (weak), D-glucuronate (weak), and glucono-1,5-lactone. Methanol, ethanol, glycerol, erythritol, and DL-lactate are not assimilated. Nitrate (delayed and weak), nitrite (delayed and weak), and L-lysine are assimilated as sole nitrogen sources. Ethylamine and cadaverine are not assimilated. Maximum growth temperature is 30 °C. Growth on 50% (w/w) glucose-yeast extract agar is negative. Growth in vitamin-free medium is positive. Starch-like substances are not produced. Urease activity is positive. Diazonium Blue B reaction is positive.

##### Additional strain examined.

China• Guizhou Prov.: Pingtang county, Sifangjing village, 25°7'N, 107°2'E, in the phylloplane of *Distyliumracemosum* Sieb. et Zucc, Feb 2023, D. Lu, NYNU 232184.

#### ﻿ Carlosrosaea wuzhiensis

Taxon classificationFungiTremellalesTrimorphomycetaceae

W.L. Gao & F.L. Hui sp. nov.

DA037D52-6CD7-56A4-A0FA-5629BBCCF230

857760

Fig. 2E, F

##### Etymology.

The specific epithet “*wuzhiensis*” refers to the geographic origin of the type strain of the species, Wuzhi Mountain.

##### Type.

China• Hainan Prov.: Sanya City, Wuzhi Mountain, 32°45'N, 113°30'E, in the phylloplane of *Glochidionzeylanicum* (Gaertn.) A. Juss, 15 Aug 2024, S.L. Lv, NYNU 24841 (holotype GDMCC 2.526^T^, preserved in a metabolically inactive state; culture ex-type PYCC 10136, preserved in a viable metabolically inactive state; GenBank: PQ568987, PQ568982).

##### Description.

On YM agar after 7 days at 20 °C, the streak culture is pale-yellow, butyrous, smooth, and glossy, with an entire margin (Fig. 2E). After 3 days in YM broth at 20 °C, cells are ovoid and ellipsoidal, 2.4–3.8 × 3.8–6.9 μm, and single; budding is polar (Fig. 2F). After 1 month at 20 °C, a ring and sediment are present. In Dalmau plate culture on CMA, pseudohyphae and hyphae are not formed. Sexual structures are not observed on PDA, CMA, or V8 agar. Ballistoconidia are not produced. Glucose fermentation is absent. The following compounds are assimilated as sole carbon sources: glucose, sucrose (weak), raffinose, melibiose (weak), galactose (weak), lactose (delayed and weak), trehalose (weak), maltose (weak), melezitose (weak), methyl-α-D-glucoside (delayed and weak), cellobiose, salicin (weak), L-sorbose (delayed and weak), L-rhamnose (delayed and weak), D-xylose (weak), L-arabinose, D-arabinose, 5-keto-D-gluconate, D-ribose (weak), glycerol (delayed and weak), ribitol, galactitol (delayed and weak), D-mannitol (delayed and weak), D-glucitol (delayed), myo-inositol (delayed and weak), DL-lactate (delayed and weak), succinate (weak), citrate, D-gluconate (weak), D-glucosamine (delayed and weak), N-acetyl-D-glucosamine (weak), 2-keto-D-gluconate (delayed and weak), D-glucuronate (weak), and glucono-1,5-lactone. Inulin, methanol, ethanol, and erythritol are not assimilated. Nitrate (delayed and weak), nitrite (delayed and weak), and L-lysine are assimilated as sole nitrogen sources. Ethylamine and cadaverine are not assimilated. Maximum growth temperature is 30 °C. Growth on 50% (w/w) glucose-yeast extract agar is negative. Growth in vitamin-free medium is positive. Starch-like substances are not produced. Urease activity is positive. Diazonium Blue B reaction is positive.

##### Additional strain examined.

China• Hainan Prov.: Sanya City, Wuzhi Mountain, 32°45'N, 113°30'E, in the phylloplane of *Glochidionzeylanicum*, 15 Aug 2024, S.L. Lv, NYNU 248116.

##### Note.

Phylogenetic analyses indicate that the closest known species to *C.wuzhiensis* sp. nov. is *C.yunnanensis*. Both form pale-yellow colonies and produce ovoid to ellipsoidal cells. However, *C.wuzhiensis* can be distinguished from *C.yunnanensis* by its ability to assimilate cellobiose, L-sorbose, D-arabinose, glycerol, galactitol, and citrate, and its inability to assimilate inulin and erythritol. Additionally, *C.wuzhiensis* can grow in a vitamin-free medium and at 25 °C, whereas *C.yunnanensis* cannot (Table 2).

## ﻿Discussion

In this study, six *Carlosrosaea* yeast strains were isolated from the surfaces of plant leaves collected across various regions of China during a yeast diversity survey conducted between 2022 and 2024. Based on phylogenetic analyses of combined ITS and LSU sequence data, along with phenotypic characterization, three novel species of *Carlosrosaea*—*C.camelliae* sp. nov., *C.glechomae* sp. nov., and *C.wuzhiensis* sp. nov.—are proposed. These findings increase the number of recognized *Carlosrosaea* species from eight to eleven. Moreover, our study revealed the presence of more than nine additional putative species within the genus that are currently cataloged but remain undescribed, in agreement with findings from previous studies (Li et al. 2020; Félix et al. 2024; Jiang et al. 2024). The existence of these uncharacterized yet documented taxa underscores the need for the formal description of species already deposited in public databases, as well as expanded sampling to address the Linnean shortfall (Félix et al. 2024).

*Carlosrosaea* species were initially thought to produce ballistoconidia, a hypothesis inferred from their original classification within the genus *Bullera* (Liu et al. 2015). However, most known *Carlosrosaea* species do not exhibit ballistoconidium formation (Félix et al. 2017; Li et al. 2020; Jiang et al. 2024). In the present study, the species were isolated using the ballistospore-fall method, despite their inability to form ballistospores. This finding suggests that the ballistospore-fall method, while selective, is not exclusive for isolating ballistoconidium-forming yeasts. The recovery of these three *Carlosrosaea* species using this method may reflect the presence of vegetative yeast cells inhabiting the phylloplane, rather than true ballistospore production.

Of the 49 plant leaf samples collected in this study, in addition to the six *Carlosrosaea* strains, 65 yeast strains belonging to 26 previously described species were isolated. These species represented the genera *Bullera*, *Derxomyces*, *Dioszegia*, *Erythrobasidium*, *Filobasidium*, *Hannaella*, *Saitozyma*, *Symmetrospora*, *Tilletiopsis*, and *Vishniacozyma*. *Symmetrospora* was the most frequently recovered genus, suggesting that *Carlosrosaea* species are relatively rare members of the phyllosphere yeast community. Nevertheless, the discovery of these three new species highlights the widespread, albeit low-abundance, distribution of *Carlosrosaea* on plant surfaces. These results emphasize the importance of extensive sampling and combined molecular and phenotypic analyses to fully uncover the global diversity of this genus.

## Supplementary Material

XML Treatment for Carlosrosaea camelliae

XML Treatment for Carlosrosaea glechomae

XML Treatment for Carlosrosaea wuzhiensis

## ﻿Additional information

### Author ORCIDs

Wan-Li Gao https://orcid.org/0009-0000-1100-7521

Chun-Yue Chai https://orcid.org/0000-0003-0284-5560

Qiu-Hong Niu https://orcid.org/0000-0003-1695-7117

Fen-Li Hui https://orcid.org/0000-0001-7928-3055

### Data availability

All of the data that support the findings of this study are available in the main text.

## References

[B1] Andrew R (2016) FigTree: Tree figure drawing tool Version 1.4.3. Institute of Evolutionary Biology, United Kingdom, University of Edinburgh Press.

[B2] Chai CY Xi ZW Niu QH Hui FL (2024) Three new species of the genus *Kockovaella* (Cuniculitremaceae, Tremellales) from the phylloplane in China. MycoKeys 110: 237–253. 10.3897/mycokeys.110.133084 39610861 PMC11602965

[B3] do Carmo-Sousa L Phaff HJ (1962) An improved method for the detection of spore discharge in the Sporobolomycetaceae. Journal of Bacteriology 83(2): 434–435. 10.1128/jb.83.2.434-435.1962 16561932 PMC277751

[B4] Félix CR Navarro HMC Paulino GVB Broetto L Landell MF (2017) *Carlosrosaeahohenbergiae* sp. nov. and *Carlosrosaeaaechmeae* sp. nov., two tremellaceous yeasts isolated from bromeliads in north-eastern Brazil. International Journal of Systematic and Evolutionary Microbiology 67(6): 1752–1757. 10.1099/ijsem.0.001856 28613149

[B5] Félix CR Navarro HMC Landell MF (2024) The Hidden Global Diversity of the Yeast Genus Carlosrosaea: A Biodiversity Databases Perspective. Yeas 41(11–12): 658–667. 10.1002/yea.3986 39623597

[B6] Fell JW Boekhout T Fonseca A Scorzetti G Statzell-Tallman A (2000) Biodiversity and systematics of basidiomycetous yeasts as determined by large-subunit rDNA D1/D2 domain sequence analysis. International Journal of Systematic and Evolutionary Microbiology 50(3): 1351–1371. 10.1099/00207713-50-3-1351 10843082

[B7] Hall TA (1999) Bioedit: A user-friendly biological sequence alignment editor and analysis program for Windows 95/98/NT. Nucleic Acids Symposium Series 41: 95–98.

[B8] Jiang YL Bao WJ Liu F Wang GS Yurkov AM Ma Q Hu ZD Chen XH Zhao WN Li AH Wang QM (2024) Proposal of one new family, seven new genera and seventy new basidiomycetous yeast species mostly isolated from Tibet and Yunnan provinces, China. Studies in Mycology 109: 57–153. 10.3114/sim.2024.109.02 39717653 PMC11663428

[B9] Katoh K Standley DM (2013) MAFFT multiple sequence alignment software version 7: Improvements in performance and usability. Molecular Biology and Evolution 30(4): 772–780. 10.1093/molbev/mst010 23329690 PMC3603318

[B10] Kurtzman CP Robnett CJ (1998) Identification and phylogeny of ascomycetous yeasts from analysis of nuclear large subunit (26S) ribosomal DNA partial sequences. Antonie van Leeuwenhoek 73(4): 331–371. 10.1023/a:1001761008817 9850420

[B11] Kurtzman CP Fell JW Boekhout T (2011) Methods for isolation, phenotypic characterization and maintenance of yeasts. In: Kurtzman CP Fell JW Boekhout T (Eds) The Yeasts – a Taxonomic Study, 5th edn., Vol. 1. Amsterdam, Elsevier, 87–110. 10.1016/B978-0-444-52149-1.00007-0

[B12] Landell MF Brandão LR Safar SVB Gomes FCO Félix CR Santos ARO Pagani DM Ramos JP Broetto L Mott T Vainstein MH Valente P Rosa CA (2015) *Bulleravrieseae* sp. nov., a tremellaceous yeast species isolated from bromeliads. International Journal of Systematic and Evolutionary Microbiology 65(8): 2466–2471. 10.1099/ijs.0.000285 25911536

[B13] Li AH Yuan FX Groenewald M Bensch K Yurkov AM Li K Han PJ Guo LD Aime MC Sampaio JP Jindamorakot S Turchetti B Inacio J Fungsin B Wang QM Bai FY (2020) Diversity and phylogeny of basidiomycetous yeasts from plant leaves and soil: Proposal of two new orders, three new families, eight new genera and one hundred and seven new species. Studies in Mycology 96: 17–140. 10.1016/j.simyco.2020.01.002 32206137 PMC7082220

[B14] Liu XZ Wang QM Göker M Groenewald M Kachalkin AV Lumbsch HT Millanes AM Wedin M Yurkov AM Boekhout T Bai FY (2015) Towards an integrated phylogenetic classification of the Tremellomycetes. Studies in Mycology 81: 85–147. 10.1016/j.simyco.2015.12.001 26955199 PMC4777781

[B15] Marques AR Resende AA Gomes FCO Santos ARO Rosa CA Duarte AA de Lemos-Filho JP Dos Santos VL (2021) Plant growth-promoting traits of yeasts isolated from the tank bromeliad *Vrieseaminarum* L.B. Smith and the effectiveness of *Carlosrosaeavrieseae* for promoting bromeliad growth. Brazilian Journal of Microbiology 52(3): 1417–1429. 10.1007/s42770-021-00496-1 33956333 PMC8324700

[B16] Ronquist F Teslenko M van der Mark P Ayres DL Darling A Höhna S Larget B Liu L Suchard MA Huelsenbeck JP (2012) MrBayes3.2: Efficient Bayesian phylogenetic inference and model choice, across a large model space. Systems Biology 61(3): 539–542. 10.1093/sysbio/sys029 PMC3329765 22357727

[B17] Scorzetti G Fell JW Fonseca A Statzell-Tallman A (2002) Systematics of basidiomycetous yeasts: A comparison of large subunit D1/D2 and internal transcribed spacer rDNA regions. FEMS Yeast Research 2(4): 495–517. 10.1016/S1567-1356(02)00128-9 12702266

[B18] Stamatakis A (2014) RAxML Version 8: A tool for phylogenetic analyses and post analyses of large phylogenies. Bioinformatics 30(9): 1312–1313. 10.1093/bioinformatics/btu033 24451623 PMC3998144

[B19] Tamura K Stecher G Kumar S (2021) MEGA11: Molecular Evolutionary Genetics Analysis Version 11. Molecular Biology and Evolution 38(7): 3022–3027. 10.1093/molbev/msab120 33892491 PMC8233496

[B20] Toome M Roberson RW Aime MC (2013) *Meredithblackwelliaeburnea* gen. et sp. nov., Kriegeriaceae fam. nov. and Kriegeriales ord. nov.—toward resolving higher-level classification in Microbotryomycetes. Mycologia 105(2): 486–495. 10.3852/12-251 23099516

[B21] Vu D Groenewald M Szöke S Cardinali G Eberhardt U Stielow B de Vries M Verkleij GJ Crous PW Boekhout T Robert V (2016) DNA barcoding analysis of more than 9 000 yeast isolates contributes to quantitative thresholds for yeast species and genera delimitation. Studies in Mycology 85: 91–105. 10.1016/j.simyco.2016.11.007 28050055 PMC5192050

[B22] White TJ Bruns T Lee S Taylor J (1990) Amplification and direct sequencing of fungal ribosomal RNA genes for phylogenetics. In: Innis MA Gelfand DH Sninsky JJ White TJ (Eds) PCR Protocols: A Guide to Methods and Applications.Academic Press, New York, 315–322. 10.1016/B978-0-12-372180-8.50042-1

